# Bounded perturbation resilience of extragradient-type methods and their applications

**DOI:** 10.1186/s13660-017-1555-0

**Published:** 2017-11-10

**Authors:** Q-L Dong, A Gibali, D Jiang, Y Tang

**Affiliations:** 1 Tianjin Key Laboratory for Advanced Signal Processing, College of Science, Civil Aviation University of China, Tianjin, 300300 P.R. China; 2 Department of Mathematics, ORT Braude College, Karmiel, 2161002 Israel; 3 Department of Mathematics, NanChang University, Nanchang, 330031 P.R. China

**Keywords:** 49J35, 58E35, 65K15, 90C47, inertial-type method, bounded perturbation resilience, extragradient method, subgradient extragradient method, variational inequality

## Abstract

In this paper we study the bounded perturbation resilience of the extragradient and the subgradient extragradient methods for solving a variational inequality (VI) problem in real Hilbert spaces. This is an important property of algorithms which guarantees the convergence of the scheme under summable errors, meaning that an inexact version of the methods can also be considered. Moreover, once an algorithm is proved to be bounded perturbation resilience, superiorization can be used, and this allows flexibility in choosing the bounded perturbations in order to obtain a superior solution, as well explained in the paper. We also discuss some inertial extragradient methods. Under mild and standard assumptions of monotonicity and Lipschitz continuity of the VI’s associated mapping, convergence of the perturbed extragradient and subgradient extragradient methods is proved. In addition we show that the perturbed algorithms converge at the rate of $O(1/t)$. Numerical illustrations are given to demonstrate the performances of the algorithms.

## Introduction

In this paper we are concerned with the variational inequality (VI) problem of finding a point $x^{\ast}$ such that

1.1 $$ \bigl\langle F\bigl(x^{\ast}\bigr),x-x^{\ast}\bigr\rangle \geq0 \quad\text{for all }x\in C, $$

where $C\subseteq\mathcal{H}$ is a nonempty, closed and convex set in a real Hilbert space $\mathcal{H}$, $\langle\cdot,\cdot\rangle$ denotes the inner product in $\mathcal{H}$, and $F:\mathcal{H}\rightarrow\mathcal{H}$ is a given mapping. This problem is a fundamental problem in optimization theory, and it captures various applications such as partial differential equations, optimal control and mathematical programming; for the theory and application of VIs and related problems, the reader is referred, for example, to the works of Ceng *et al*. [1], Zegeye *et al*. [2], the papers of Yao *et al*. [3–5] and the many references therein.

Many algorithms for solving VI (1.1) are projection algorithms that employ projections onto the feasible set *C* of VI (1.1), or onto some related set, in order to reach iteratively a solution. Korpelevich [6] and Antipin [7] proposed an algorithm for solving (1.1), known as the *extragradient method*, see also Facchinei and Pang [8, Chapter 12]. In each iteration of the algorithm, in order to get the next iterate $x^{k+1}$, two orthogonal projections onto *C* are calculated according to the following iterative step. Given the current iterate $x^{k}$, calculate

1.2 $$ \left \{ \textstyle\begin{array}{l} y^{k}=P_{C}(x^{k}-\gamma_{k}F(x^{k})) , \\ x^{k+1}=P_{C}(x^{k}-\gamma_{k}F(y^{k})),\end{array}\displaystyle \right . $$

where $\gamma_{k}\in(0,1/L)$, and *L* is the Lipschitz constant of *F*, or $\gamma_{k}$ is updated by the following adaptive procedure:

1.3 $$ \gamma_{k}\big\Vert F\bigl(x^{k}\bigr)-F\bigl(y^{k} \bigr)\big\Vert \leq\mu\big\Vert x^{k}-y^{k}\big\Vert ,\quad\mu\in(0,1). $$

In the extragradient method there is the need to calculate twice the orthogonal projection onto *C* in each iteration. In case that the set *C* is simple enough so that projections onto it can be easily computed, this method is particularly useful; but if *C* is a general closed and convex set, a minimal distance problem has to be solved (twice) in order to obtain the next iterate. This might seriously affect the efficiency of the extragradient method. Hence, Censor *et al*. in [9–11] presented a method called the *subgradient extragradient method*, in which the second projection (1.2) onto *C* is replaced by a specific subgradient projection which can be easily calculated. The iterative step has the following form:

1.4 $$ \left \{ \textstyle\begin{array}{l} y^{k}=P_{C}(x^{k}-\gamma F(x^{k})) , \\ x^{k+1}=P_{T_{k}}(x^{k}-\gamma F(y^{k})),\end{array}\displaystyle \right . $$

where $T_{k}$ is the set defined as

1.5 $$ T_{k}:=\bigl\{ w\in\mathcal{H}\mid \bigl\langle \bigl( x^{k}-\gamma F\bigl(x^{k}\bigr) \bigr) -y^{k},w-y^{k} \bigr\rangle \leq0\bigr\} , $$

and $\gamma\in(0,1/L)$.

In this manuscript we prove that the above methods, the extragradient and the subgradient extragradient methods, are bounded perturbation resilient, and the perturbed methods have the convergence rate of $O(1/t)$. This means that that will show that an inexact version of the algorithms allows incorporating summable errors that also converge to a solution of VI (1.1) and, moreover, their superiorized version can be introduced by choosing the perturbations. In order to obtain a superior solution with respect to some new objective function, for example, by choosing the norm, we can obtain a solution to VI (1.1) which is closer to the origin.

Our paper is organized as follows. In Section 2 we present the preliminaries. In Section 3 we study the convergence of the extragradient method with outer perturbations. Later, in Section 4, the bounded perturbation resilience of the extragradient method as well as the construction of the inertial extragradient methods are presented.

In the spirit of the previous sections, in Section 5 we study the convergence of the subgradient extragradient method with outer perturbations, show its bounded perturbation resilience and the construction of the inertial subgradient extragradient methods. Finally, in Section 6 we present numerical examples in signal processing which demonstrate the performances of the perturbed algorithms.

## Preliminaries

Let $\mathcal{H}$ be a real Hilbert space with the inner product $\langle\cdot ,\cdot\rangle$ and the induced norm $\Vert\cdot\Vert$, and let *D* be a nonempty, closed and convex subset of $\mathcal{H}$. We write $x^{k}\rightharpoonup x$ to indicate that the sequence $\{ x^{k} \} _{k=0}^{\infty}$ converges weakly to *x* and $x^{k}\rightarrow x$ to indicate that the sequence $\{ x^{k} \} _{k=0}^{\infty}$ converges strongly to *x*. Given a sequence $\{ x^{k} \} _{k=0}^{\infty}$, denote by $\omega_{w}(x^{k})$ its weak *ω*-limit set, that is, any $x\in\omega_{w}(x^{k})$ such that there exists a subsequence $\{ x^{k_{j}} \} _{j=0}^{\infty}$ of $\{ x^{k} \} _{k=0}^{\infty}$ which converges weakly to *x*.

For each point $x\in\mathcal{H}$, there exists a unique nearest point in *D* denoted by $P_{D}(x)$. That is,

2.1 $$ \bigl\Vert x-P_{D} ( x ) \bigr\Vert \leq \Vert x-y \Vert \quad\text{for all }y\in D. $$

The mapping $P_{D}:\mathcal{H}\rightarrow D$ is called the metric projection of $\mathcal{H}$ onto *D*. It is well known that $P_{D}$ is a *nonexpansive mapping* of $\mathcal{H}$ onto *D*, *i.e*., and even *firmly nonexpansive mapping. This is captured in the next lemma.*

### Lemma 2.1

*For any* $x,y\in\mathcal{H}$ *and* $z\in D$, *it holds*

- $\Vert P_{D}(x)-P_{D}(y)\Vert^{2}\leq\Vert x-y\Vert$;
- $\Vert P_{D}(x)-z\Vert^{2}\leq\Vert x-z\Vert^{2}-\Vert P_{D}(x)-x\Vert^{2}$.

The characterization of the metric projection $P_{D}$ [12, Section 3] is given by the following two properties in this lemma.

### Lemma 2.2

*Given* $x\in\mathcal{H}$ *and* $z\in D$. *Then* $z=P_{D} ( x ) $ *if and only if*

2.2 $$ P_{D}(x)\in D $$

*and*

2.3 $$ \bigl\langle x-P_{D} ( x ) ,P_{D} ( x ) -y \bigr\rangle \geq0\quad\textit{for all }x\in\mathcal{H},y\in D. $$

### Definition 2.3

The normal cone of *D* at $v\in D$ denoted by $N_{D} ( v ) $ is defined as

2.4 $$ N_{D} ( v ) :=\bigl\{ d\in\mathcal{H}\mid \langle d,y-v \rangle\leq0 \text{ for all }y\in D\bigr\} . $$

### Definition 2.4

Let $B:\mathcal{H}\rightrightarrows2^{\mathcal{H}}$ be a point-to-set operator defined on a real Hilbert space $\mathcal{H}$. The operator *B* is called a maximal monotone operator if *B* is monotone, *i.e*.,

2.5 $$ \langle u-v,x-y \rangle\geq0\quad\text{for all }u\in B(x)\text{ and }v\in B(y), $$

and the graph $G(B)$ of *B*,

2.6 $$ G(B):= \bigl\{ ( x,u ) \in\mathcal{H}\times\mathcal{H}\mid u\in B(x) \bigr\} , $$

is not properly contained in the graph of any other monotone operator.

Based on Rockafellar [13, Theorem 3], a monotone mapping *B* is maximal if and only if, for any $( x,u ) \in\mathcal{H}\times \mathcal{H}$, if $\langle u-v,x-y \rangle\geq0$ for all $( v,y ) \in G(B)$, then it follows that $u\in B(x)$.

### Definition 2.5

The subdifferential set of a convex function *c* at a point *x* is defined as

2.7 $$ \partial c(x):=\bigl\{ \xi\in\mathcal{H}\mid c(y)\geq c(x)+\langle\xi ,y-x\rangle \text{ for all }y\in\mathcal{H}\bigr\} . $$

For $z\in\mathcal{H}$, take any $\xi\in\partial c(z)$ and define

2.8 $$ T ( z ) := \bigl\{ w\in\mathcal{H}\mid c(z)+\langle\xi ,w-z\rangle \leq0 \bigr\} . $$

This is a half-space, the bounding hyperplane of which separates the set *D* from the point *z* if $\xi\neq0$; otherwise $T(z)=\mathcal{H}$; see, *e.g*., [14, Lemma 7.3].

### Lemma 2.6

([15])

*Let* *D* *be a nonempty*, *closed and convex subset of a Hilbert space* $\mathcal{H}$. *Let* $\{x^{k}\}_{k=0}^{\infty}$ *be a bounded sequence which satisfies the following properties*:

- *every limit point of* $\{x^{k}\}_{k=0}^{\infty}$ *lies in* *D*;
- $\lim_{n\rightarrow\infty}\Vert x^{k}-x\Vert$ *exists for every* $x\in D$.

*Then* $\{x^{k}\}_{k=0}^{\infty}$ *converges to a point in* *D*.

### Lemma 2.7

*Assume that* $\{a_{k}\}_{k=0}^{\infty}$ *is a sequence of nonnegative real numbers such that*

2.9 $$ a_{k+1}\leq(1+\gamma_{k})a_{k}+ \delta_{k},\quad\forall k\geq0, $$

*where the nonnegative sequences* $\{\gamma_{k}\}_{k=0}^{\infty}$ *and* $\{\delta_{k}\}_{k=0}^{\infty}$ *satisfy* $\sum_{k=0}^{\infty}\gamma _{k}<+\infty$ *and* $\sum_{k=0}^{\infty}\delta_{k}<+\infty$, *respectively*. *Then* $\lim_{k\rightarrow\infty}a_{k}$ *exists*.

## The extragradient method with outer perturbations

In order to discuss the convergence of the extragradient method with outer perturbations, we make the following assumptions.

### Condition 3.1

The solution set of (1.1), denoted by $\mathit{SOL}(C,F)$, is nonempty.

### Condition 3.2

The mapping *F* is *monotone* on *C*, *i.e*.,

3.1 $$ \bigl\langle F(x)-F(y),x-y\bigr\rangle \geq0,\quad\forall x,y\in C. $$

### Condition 3.3

The mapping *F* is *Lipschitz continuous* on *C* with the Lipschitz constant $L>0$, *i.e*.,

3.2 $$ \big\Vert F(x)-F(y)\big\Vert \leq L\Vert x-y\Vert,\quad\forall x,y\in C. $$

Observe that while Censor *et al*. in [10, Theorem 3.1] showed the weak convergence of the extragradient method (1.2) in Hilbert spaces for a fixed step size $\gamma_{k}=\gamma\in(0,1/L)$, this can be easily improved in case that the adaptive rule (1.3) is used. The next theorem shows this, and its proof can easily be derived by following similar lines of the proof of [10, Theorem 3.1].

### Theorem 3.4

*Assume that Conditions* 3.1-3.3 *hold*. *Then any sequence* $\{x^{k}\}_{k=0}^{\infty}$ *generated by the extragradient method* (1.2) *with the adaptive rule* (1.3) *weakly converges to a solution of the variational inequality* (1.1).

Denote $e_{i}^{k}:=e_{i}(x^{k})$, $i=1,2$. The sequences of perturbations $\{e_{i}^{k}\}_{k=0}^{\infty}$, $i=1,2$, are assumed to be summable, *i.e*.,

3.3 $$ \sum_{k=0}^{\infty}\big\Vert e_{i}^{k}\big\Vert < +\infty,\quad i=1,2. $$

Now we consider the extragradient method with outer perturbations.

### Algorithm 3.5

The extragradient method with outer perturbations:

*Step 0*: Select a starting point $x^{0}\in C$ and set $k=0$.

*Step 1*: Given the current iterate $x^{k}$, compute

3.4 $$ y^{k}=P_{C}\bigl(x^{k}- \gamma_{k}F\bigl(x^{k}\bigr)+e_{1} \bigl(x^{k}\bigr)\bigr), $$

where $\gamma_{k}=\sigma\rho^{m_{k}}$, $\sigma>0$, $\rho\in(0,1)$ and $m_{k}$ is the smallest nonnegative integer such that (see [16])

3.5 $$ \gamma_{k}\big\Vert F\bigl(x^{k}\bigr)-F\bigl(y^{k} \bigr)\big\Vert \leq\mu\big\Vert x^{k}-y^{k}\big\Vert ,\quad\mu\in(0,1). $$

Calculate the next iterate

3.6 $$ x^{k+1}=P_{C}\bigl(x^{k}-\gamma_{k}F \bigl(y^{k}\bigr)+e_{2}\bigl(x^{k}\bigr)\bigr). $$

*Step 2*: If $x^{k}=y^{k}$, then stop. Otherwise, set $k\leftarrow(k+1)$ and return to *Step 1*.

### Convergence analysis

#### Lemma 3.6

([17])

*The Armijo*-*like search rule* (3.5) *is well defined*. *Besides*, $\underline{\gamma}\leq\gamma_{k}\leq\sigma$, *where* $\underline{\gamma}=\min\{\sigma,\frac{\mu\rho}{L}\}$.

#### Theorem 3.7

*Assume that Conditions* 3.1-3.3 *hold*. *Then the sequence* $\{x^{k}\}_{k=0}^{\infty}$ *generated by Algorithm* 3.5 *converges weakly to a solution of the variational inequality* (1.1).

#### Proof

Take $x^{*}\in \mathit{SOL}(C,F)$. From (3.6) and Lemma 2.1(ii), we have

3.7 $$ \begin{aligned}[b] \big\| x^{k+1}-x^{*} \big\| ^{2}\leq{}&\big\| x^{k}-\gamma_{k} F\bigl(y^{k} \bigr)+e_{2}^{k}-x^{*}\big\| ^{2}- \big\| x^{k}- \gamma_{k} F\bigl(y^{k}\bigr)+e_{2}^{k}-x^{k+1} \big\| ^{2} \\ ={}&\big\| x^{k}-x^{*}\big\| ^{2}-\big\| x^{k}-x^{k+1} \big\| ^{2}+2\gamma_{k}\bigl\langle F\bigl(y^{k} \bigr),x^{*}-x^{k+1}\bigr\rangle \\ &-2\bigl\langle e_{2}^{k},x^{*}-x^{k+1}\bigr\rangle . \end{aligned} $$

From the Cauchy-Schwarz inequality and the mean value inequality, it follows

3.8 $$ \begin{aligned}[b] -2\bigl\langle e_{2}^{k},x^{*}-x^{k+1} \bigr\rangle &\leq2\big\| e_{2}^{k}\big\| \big\| x^{k+1}-x^{*}\big\| \\ &\leq\big\| e_{2}^{k}\big\| +\big\| e_{2}^{k}\big\| \big\| x^{k+1}-x^{*}\big\| ^{2}. \end{aligned} $$

Using $x^{*}\in \mathit{SOL}(C,F)$ and the monotone property of *F*, we have $\langle y^{k}-x^{*},F(y^{k})\rangle\geq0$ and, consequently, we get

3.9 $$ 2\gamma_{k}\bigl\langle F\bigl(y^{k} \bigr),x^{*}-x^{k+1}\bigr\rangle \leq2\gamma_{k}\bigl\langle F \bigl(y^{k}\bigr),y^{k}-x^{k+1}\bigr\rangle . $$

Thus, we have

3.10 $$ \begin{aligned}[b] &{-}\big\| x^{k}-x^{k+1} \big\| ^{2}+2\gamma_{k}\bigl\langle F\bigl(y^{k} \bigr),x^{*}-x^{k+1}\bigr\rangle \\ &\quad\leq-\big\| x^{k}-x^{k+1}\big\| ^{2}+2\gamma_{k} \bigl\langle F\bigl(y^{k}\bigr),y^{k}-x^{k+1}\bigr\rangle \\ &\quad=-\big\| x^{k}-y^{k}\big\| ^{2}-\big\| y^{k}-x^{k+1} \big\| ^{2} \\ &\quad\quad{}+2\bigl\langle x^{k}-\gamma_{k}F\bigl(y^{k} \bigr)-y^{k},x^{k+1}-y^{k}\bigr\rangle , \end{aligned} $$

where the equality comes from

3.11 $$ -\big\| x^{k}-x^{k+1}\big\| ^{2}=- \big\| x^{k}-y^{k}\big\| ^{2}-\big\| y^{k}-x^{k+1} \big\| ^{2}-2\bigl\langle x^{k}-y^{k},y^{k}-x^{k+1} \bigr\rangle . $$

Using $x^{k+1}\in C$, the definition of $y^{k}$ and Lemma 2.2, we have

3.12 $$ \bigl\langle y^{k}-x^{k}+ \gamma_{k}F\bigl(x^{k}\bigr)-e_{1}^{k},x^{k+1}-y^{k} \bigr\rangle \geq0. $$

So, we obtain

3.13 $$ \begin{aligned}[b] &2\bigl\langle x^{k}- \gamma_{k}F\bigl(y^{k}\bigr)-y^{k},x^{k+1}-y^{k} \bigr\rangle \\ &\quad\leq2\gamma_{k}\bigl\langle F\bigl(x^{k}\bigr)-F \bigl(y^{k}\bigr),x^{k+1}-y^{k}\bigr\rangle -2\bigl\langle e_{1}^{k},x^{k+1}-y^{k}\bigr\rangle \\ &\quad\leq2\gamma_{k}\big\| F\bigl(x^{k}\bigr)-F\bigl(y^{k} \bigr)\big\| \big\| x^{k+1}-y^{k}\big\| +2\big\| e_{1}^{k}\big\| \big\| x^{k+1}-y^{k}\big\| \\ &\quad\leq2\mu\big\| x^{k}-y^{k}\big\| \big\| x^{k+1}-y^{k} \big\| +\big\| e_{1}^{k}\big\| +\big\| e_{1}^{k}\big\| \big\| x^{k+1}-y^{k}\big\| ^{2} \\ &\quad\leq\mu\big\| x^{k}-y^{k}\big\| ^{2}+\mu \big\| x^{k+1}-y^{k}\big\| ^{2}+\big\| e_{1}^{k} \big\| +\big\| e_{1}^{k}\big\| \big\| x^{k+1}-y^{k} \big\| ^{2} \\ &\quad=\mu\big\| x^{k}-y^{k}\big\| ^{2}+\bigl(\mu+\big\| e_{1}^{k}\big\| \bigr)\big\| x^{k+1}-y^{k} \big\| ^{2}+\big\| e_{1}^{k}\big\| . \end{aligned} $$

From (3.3), it follows

3.14 $$ \lim_{k\rightarrow\infty}\big\| e_{i}^{k}\big\| =0, \quad i=1,2. $$

Therefor, we assume $\|e_{1}^{k}\|\in[0,1- \mu-\nu)$ and $\|e_{2}^{k}\|\in [0,1/2)$, $k\geq0$, where $\nu\in(0,1-\mu)$. So, using (3.13), we get

3.15 $$ 2\bigl\langle x^{k}-\gamma_{k}F \bigl(y^{k}\bigr)-y^{k},x^{k+1}-y^{k}\bigr\rangle \leq\mu\big\| x^{k}-y^{k}\big\| ^{2}+(1-\nu) \big\| x^{k+1}-y^{k}\big\| ^{2}+\big\| e_{1}^{k} \big\| . $$

Combining (3.7)-(3.10) and (3.15), we obtain

3.16 $$ \begin{aligned}[b] \big\| x^{k+1}-x^{*}\big\| ^{2} \leq{}&\big\| x^{k}-x^{*}\big\| ^{2}- (1-\mu )\big\| x^{k}-y^{k} \big\| ^{2}-\nu\big\| x^{k+1}-y^{k}\big\| ^{2} \\ &+\big\| e^{k}\big\| +\big\| e_{2}^{k}\big\| \big\| x^{k+1}-x^{*}\big\| ^{2}, \end{aligned} $$

where

3.17 $$ \big\| e^{k}\big\| :=\big\| e_{1}^{k}\big\| + \big\| e_{2}^{k}\big\| . $$

From (3.16), it follows

3.18 $$ \begin{aligned}[b] \big\| x^{k+1}-x^{*} \big\| ^{2}&\leq\frac{1}{1-\|e_{2}^{k}\|}\big\| x^{k}-x^{*}\big\| ^{2} - \frac{1-\mu }{1-\|e_{2}^{k}\|}\big\| x^{k}-y^{k}\big\| ^{2} \\ &\quad-\frac{\nu}{1-\|e_{2}^{k}\|}\big\| x^{k+1}-y^{k}\big\| ^{2}+ \frac{\|e^{k}\|}{1-\| e_{2}^{k}\|}. \end{aligned} $$

Since $\|e_{2}^{k}\|\in[0,1/2)$, $k\geq0$, we get

3.19 $$ 1\leq\frac{1}{1-\|e_{2}^{k}\|}\leq1+2\big\| e_{2}^{k}\big\| < 2. $$

So, from (3.18), we have

3.20 $$ \begin{aligned}[b] \big\| x^{k+1}-x^{*} \big\| ^{2}\leq{}&\bigl(1+2\big\| e_{2}^{k}\big\| \bigr) \big\| x^{k}-x^{*}\big\| ^{2}-(1-\mu)\big\| x^{k}-y^{k} \big\| ^{2} \\ &-\nu\big\| x^{k+1}-y^{k}\big\| ^{2}+2\big\| e^{k} \big\| \\ \leq{}&\bigl(1+2\big\| e_{2}^{k}\big\| \bigr)\big\| x^{k}-x^{*} \big\| ^{2}+2\big\| e^{k}\big\| . \end{aligned} $$

Using (3.3) and Lemma 2.7, we get the existence of $\lim_{k\rightarrow\infty}\|x^{k}-x^{*}\|^{2}$ and then the boundedness of $\{x^{k}\}_{k=0}^{\infty}$. From (3.20), it follows

3.21 $$ (1-\mu)\big\| x^{k}-y^{k}\big\| ^{2}+\nu \big\| x^{k+1}-y^{k}\big\| ^{2}\leq\bigl(1+2 \big\| e_{2}^{k}\big\| \bigr)\big\| x^{k}-x^{*}\big\| ^{2}-\big\| x^{k+1}-x^{*}\big\| ^{2}+2\big\| e^{k}\big\| , $$

which means that

3.22 $$ \sum_{k=0}^{\infty}\big\| x^{k}-y^{k}\big\| ^{2} < +\infty\quad\hbox{and}\quad \sum_{k=0}^{\infty}\big\| x^{k+1}-y^{k} \big\| ^{2} < +\infty. $$

Thus, we obtain

3.23 $$ \lim_{k\rightarrow\infty}\big\| x^{k}-y^{k} \big\| =0\quad\hbox{and}\quad \lim_{k\rightarrow\infty}\big\| x^{k+1}-y^{k} \big\| =0, $$

and consequently,

3.24 $$ \lim_{k\rightarrow\infty}\big\| x^{k+1}-x^{k} \big\| =0. $$

Now, we are to show $\omega_{w}(x^{k})\subseteq \mathit{SOL}(C,F)$. Due to the boundedness of $\{x^{k}\}_{k=0}^{\infty}$, it has at least one weak accumulation point. Let $\hat{x}\in\omega_{w}(x^{k})$. Then there exists a subsequence $\{x^{k_{i}}\} _{i=0}^{\infty}$ of $\{x^{k}\}_{k=0}^{\infty}$ which converges weakly to *x̂*. From (3.23), it follows that $\{y^{k_{i}}\}_{i=0}^{\infty}$ also converges weakly to *x̂*.

We will show that *x̂* is a solution of the variational inequality (1.1). Let

3.25 $$ A(v)=\left \{ \textstyle\begin{array}{l@{\quad}l} F(v)+N_{C} ( v ) , & v\in C, \\ \emptyset, & v\notin C,\end{array}\displaystyle \right . $$

where $N_{C}(v)$ is the normal cone of *C* at $v\in C$. It is known that *A* is a maximal monotone operator and $A^{-1}(0)=\mathit{SOL}(C,F)$. If $(v,w)\in G(A)$, then we have $w-F(v)\in N_{C}(v)$ since $w\in A(v)=F(v)+N_{C}(v)$. Thus it follows that

3.26 $$ \bigl\langle w-F(v),v-y\bigr\rangle \geq0,\quad y\in C. $$

Since $y^{k_{i}}\in C$, we have

3.27 $$ \bigl\langle w-F(v),v-y^{k_{i}}\bigr\rangle \geq0. $$

On the other hand, by the definition of $y^{k}$ and Lemma 2.2, it follows that

3.28 $$ \bigl\langle x^{k}-\gamma_{k} F \bigl(x^{k}\bigr)+e_{1}^{k}-y^{k}, y^{k}-v\bigr\rangle \geq0, $$

and consequently,

3.29 $$ \biggl\langle \frac{y^{k}-x^{k}}{\gamma_{k}}+F\bigl(x^{k} \bigr),v-y^{k} \biggr\rangle -\frac{1}{\gamma_{k}}\bigl\langle e_{1}^{k}, v-y^{k} \bigr\rangle \geq0. $$

Hence we have

3.30 $$ \begin{aligned}[b] \bigl\langle w,v-y^{k_{i}} \bigr\rangle \geq{}&\bigl\langle F(v),v-y^{k_{i}}\bigr\rangle \\ \geq{}&\bigl\langle F(v),v-y^{k_{i}}\bigr\rangle - \biggl\langle \frac{y^{k_{i}}-x^{k_{i}}}{\gamma_{{k_{i}}}}+F\bigl(x^{k_{i}}\bigr),v-y^{k_{i}} \biggr\rangle + \frac{1}{\gamma _{k_{i}}}\bigl\langle e_{1}^{k_{i}}, v-y^{k_{i}} \bigr\rangle \\ ={}&\bigl\langle F(v)-F\bigl(y^{k_{i}}\bigr),v-y^{k_{i}}\bigr\rangle +\bigl\langle F\bigl(y^{k_{i}}\bigr)-F\bigl(x^{k_{i}} \bigr),v-y^{k_{i}}\bigr\rangle \\ &- \biggl\langle \frac{y^{k_{i}}-x^{k_{i}}}{\gamma_{k_{i}}},v-y^{k_{i}} \biggr\rangle + \frac{1}{\gamma_{k_{i}}}\bigl\langle e_{1}^{k_{i}}, v-y^{k_{i}} \bigr\rangle \\ \geq{}&\bigl\langle F\bigl(y^{k_{i}}\bigr)-F\bigl(x^{k_{i}} \bigr),v-y^{k_{i}}\bigr\rangle - \biggl\langle \frac{y^{k_{i}}-x^{k_{i}}}{\gamma_{k_{i}}},v-y^{k_{i}} \biggr\rangle +\frac{1}{\gamma_{k_{i}}} \bigl\langle e_{1}^{k_{i}}, v-y^{k_{i}} \bigr\rangle , \end{aligned} $$

which implies

3.31 $$ \bigl\langle w,v-y^{k_{i}}\bigr\rangle \geq\bigl\langle F \bigl(y^{k_{i}}\bigr)-F\bigl(x^{k_{i}}\bigr),v-y^{k_{i}}\bigr\rangle - \biggl\langle \frac{y^{k_{i}}-x^{k_{i}}}{\gamma_{k_{i}}},v-y^{k_{i}} \biggr\rangle +\frac{1}{\gamma_{k_{i}}}\bigl\langle e_{1}^{k_{i}}, v-y^{k_{i}} \bigr\rangle . $$

Taking the limit as $i\rightarrow\infty$ in the above inequality and using Lemma 3.6, we obtain

3.32 $$ \langle w,v-\hat{x}\rangle\geq0. $$

Since *A* is a maximal monotone operator, it follows that $\hat{x}\in A^{-1}(0) = \mathit{SOL}(C,F)$. So, $\omega_{w}(x^{k})\subseteq \mathit{SOL}(C,F)$.

Since $\lim_{k\rightarrow\infty}\|x^{k}-x^{*}\|$ exists and $\omega_{w}(x^{k})\subseteq \mathit{SOL}(C,F)$, using Lemma 2.6, we conclude that $\{x^{k}\}_{k=0}^{\infty}$ weakly converges to a solution of the variational inequality (1.1). This completes the proof. □

### Convergence rate

Nemirovski [18] and Tseng [19] proved the $O(1/t) $ convergence rate of the extragradient method. In this subsection, we present the convergence rate of Algorithm 3.5.

#### Theorem 3.8

*Assume that Conditions* 3.1-3.3 *hold*. *Let the sequences* $\{x^{k}\}_{k=0}^{\infty}$ *and* $\{y^{k}\}_{k=0}^{\infty}$ *be generated by Algorithm* 3.5. *For any integer* $t>0$, *we have* $y_{t}\in C$ *which satisfies*

3.33 $$ \begin{aligned}\bigl\langle F(x),y_{t}-x\bigr\rangle \leq \frac{1}{2 \Upsilon _{t}}\bigl(\big\Vert x-x^{0}\big\Vert ^{2}+M(x) \bigr),\quad\forall x\in C, \end{aligned} $$

*where*

3.34 $$ y_{t}=\frac{1}{\Upsilon_{t}}\sum _{k=0}^{t}\gamma_{k}y^{k},\quad \Upsilon _{t}=\sum_{k=0}^{t} \gamma_{k} $$

*and*

3.35 $$ M(x)=\sup_{k}\bigl\{ \max\bigl\{ \big\| x^{k+1}-y^{k} \big\| ,\big\| x^{k+1}-x\big\| \bigr\} \bigr\} \sum_{k=0}^{\infty } \big\Vert e^{k}\big\Vert . $$

#### Proof

Take arbitrarily $x\in C$. From Conditions 3.2 and 3.3, we have

3.36 $$ \begin{aligned}[b] &{-}\big\| x^{k}-x^{k+1} \big\| ^{2}+2\gamma_{k}\bigl\langle F\bigl(y^{k} \bigr),x-x^{k+1}\bigr\rangle \\ &\quad=-\big\| x^{k}-x^{k+1}\big\| ^{2}+2\gamma_{k} \bigl[\bigl\langle F\bigl(y^{k}\bigr)-F(x),x-y^{k}\bigr\rangle +\bigl\langle F(x),x-y^{k}\bigr\rangle \\ &\quad\quad{}+\bigl\langle F\bigl(y^{k}\bigr),y^{k}-x^{k+1} \bigr\rangle \bigr] \\ &\quad\leq-\big\| x^{k}-x^{k+1}\big\| ^{2}+2\gamma_{k} \bigl[\bigl\langle F(x),x-y^{k}\bigr\rangle +\bigl\langle F \bigl(y^{k}\bigr),y^{k}-x^{k+1}\bigr\rangle \bigr] \\ &\quad=-\big\| x^{k}-y^{k}\big\| ^{2}-\big\| y^{k}-x^{k+1} \big\| ^{2}+2\gamma_{k}\bigl\langle F(x),x-y^{k}\bigr\rangle \\ &\quad\quad{}+2\bigl\langle x^{k}-\gamma_{k}F\bigl(y^{k} \bigr)-y^{k},x^{k+1}-y^{k}\bigr\rangle . \end{aligned} $$

By (3.6) and Lemma 2.2, we get

3.37 $$ \begin{aligned}[b] &2\bigl\langle x^{k}- \gamma_{k}F\bigl(y^{k}\bigr)-y^{k},x^{k+1}-y^{k} \bigr\rangle \\ &\quad=2\bigl\langle x^{k}-\gamma_{k}F\bigl(x^{k} \bigr)+e_{1}^{k}-y^{k},x^{k+1}-y^{k} \bigr\rangle -2\bigl\langle e_{1}^{k},x^{k+1}-y^{k} \bigr\rangle \\ &\quad\quad{}+2\gamma_{k}\bigl\langle F\bigl(x^{k}\bigr)-F \bigl(y^{k}\bigr),x^{k+1}-y^{k}\bigr\rangle \\ &\quad\leq-2\bigl\langle e_{1}^{k},x^{k+1}-y^{k} \bigr\rangle +2\gamma_{k}\bigl\langle F\bigl(x^{k}\bigr)-F \bigl(y^{k}\bigr),x^{k+1}-y^{k}\bigr\rangle \\ &\quad\leq2\big\| e_{1}^{k}\big\| \big\| x^{k+1}-y^{k}\big\| +2 \mu\big\| x^{k}-y^{k}\big\| \big\| x^{k+1}-y^{k}\big\| \\ &\quad\leq2\big\| e_{1}^{k}\big\| \big\| x^{k+1}-y^{k}\big\| + \mu^{2}\big\| x^{k}-y^{k}\big\| ^{2}+ \big\| x^{k+1}-y^{k}\big\| ^{2}. \end{aligned} $$

Identifying $x^{*}$ with *x* in (3.7) and (3.8), and combining (3.36) and (3.37), we get

3.38 $$ \begin{aligned}[b] &\big\| x^{k+1}-x\big\| ^{2} \\ &\quad\leq\big\| x^{k}-x\big\| ^{2}+2\big\| e_{1}^{k}\big\| \big\| x^{k+1}-y^{k}\big\| -\bigl(1-\mu^{2}\bigr) \big\| x^{k}-y^{k}\big\| ^{2} \\ &\quad\quad{}+2\big\| e_{2}^{k}\big\| \big\| x^{k+1}-x\big\| +2 \gamma_{k}\bigl\langle F(x),x-y^{k}\bigr\rangle \\ &\quad\leq\big\| x^{k}-x\big\| ^{2}+2\big\| e_{1}^{k}\big\| \big\| x^{k+1}-y^{k}\big\| +2\big\| e_{2}^{k}\big\| \big\| x^{k+1}-x\big\| \\ &\quad\quad{}+2\gamma_{k}\bigl\langle F(x),x-y^{k}\bigr\rangle . \end{aligned} $$

Thus, we have

3.39 $$ \begin{aligned}[b] &\gamma_{k} \bigl\langle F(x),y^{k}-x\bigr\rangle \\ &\quad\leq\frac{1}{2}\bigl(\big\| x^{k}-x\big\| ^{2}- \big\| x^{k+1}-x\big\| ^{2}\bigr)+\big\| e_{1}^{k}\big\| \big\| x^{k+1}-y^{k}\big\| +\big\| e_{2}^{k}\big\| \big\| x^{k+1}-x\big\| \\ &\quad\leq\frac{1}{2}\bigl(\big\| x^{k}-x\big\| ^{2}- \big\| x^{k+1}-x\big\| ^{2}\bigr)+M^{\prime}(x)\big\| e^{k}\big\| , \end{aligned} $$

where $M^{\prime}(x)=\sup_{k}\{\max\{\|x^{k+1}-y^{k}\|,\|x^{k+1}-x\|\}\} <+\infty$. Summing inequality (3.39) over $k = 0,\ldots, t$, we obtain

3.40 $$ \begin{aligned}[b] \Biggl\langle F(x),\sum _{k=0}^{t}\gamma_{k}y^{k}- \Biggl( \sum_{k=0}^{t}\gamma_{k} \Biggr)x \Biggr\rangle &\leq\frac{1}{2}\big\| x^{0}-x\big\| ^{2}+ \frac{M^{\prime }(x)}{2}\sum_{k=0}^{t} \big\| e^{k}\big\| \\ &=\frac{1}{2}\big\| x^{0}-x\big\| ^{2}+\frac{1}{2}M(x). \end{aligned} $$

Using the notations of $\Upsilon_{t}$ and $y^{t}$ in the above inequality, we derive

3.41 $$ \bigl\langle F(x),y_{t}-x\bigr\rangle \leq \frac{1}{2\Upsilon_{t}}\bigl(\big\| x-x^{0}\big\| ^{2}+M(x)\bigr),\quad\forall x\in C. $$

The proof is complete. □

#### Remark 3.9

From Lemma 3.6, it follows

3.42 $$ \Upsilon_{t}\geq(t+1)\underline{\gamma}, $$

thus Algorithm 3.5 has $O(1/t)$ convergence rate. In fact, for any bounded subset $D \subset C$ and given accuracy $\epsilon> 0$, our algorithm achieves

3.43 $$ \bigl\langle F(x),y_{t}-x\bigr\rangle \leq\epsilon,\quad \forall x\in D $$

in at most

3.44 $$ t= \biggl[\frac{m}{2\underline{\gamma}\epsilon} \biggr] $$

iterations, where $y_{t}$ is defined by (3.34) and $m=\sup\{\| x-x^{0}\|^{2}+M(x)\mid x\in D\}$.

## The bounded perturbation resilience of the extragradient method

In this section, we prove the bounded perturbation resilience (BPR) of the extragradient method. This property is fundamental for the application of the superiorization methodology (SM) to them.

The superiorization methodology of [20–22], which originates in the papers by Butnariu, Reich and Zaslavski [23–25], is intended for constrained minimization (CM) problems of the form

4.1 $$ \min\bigl\{ \phi(x) \mid x\in\Psi\bigr\} , $$

where $\phi:H\rightarrow\mathbb{R}$ is an objective function and $\Psi \subseteq H$ is the solution set of another problem. Here, we assume $\Psi\neq \emptyset$ throughout this paper. Assume that the set Ψ is a closed convex subset of a Hilbert space *H*, the minimization problem (4.1) becomes a standard CM problem. Here, we are interested in the case wherein Ψ is the solution set of another CM of the form

4.2 $$ \min \bigl\{ f(x)\mid x\in\Omega\bigr\} , $$

*i.e*., we wish to look at

4.3 $$ \Psi:=\bigl\{ x^{\ast}\in\Omega\mid f\bigl(x^{\ast}\bigr)\leq f(x) \mid\hbox{for all }x\in\Omega\bigr\} $$

provided that Ψ is nonempty. If *f* is differentiable, and let $F=\nabla f$, then CM (4.2) equals the following variational inequality: to find a point $x^{\ast}\in C$ such that

4.4 $$ \bigl\langle F\bigl(x^{\ast}\bigr),x-x^{\ast}\bigr\rangle \geq0, \quad\forall x\in C. $$

The superiorization methodology (SM) strives not to solve (4.1) but rather the task is to find a point in Ψ which is superior, *i.e*., has a lower, but not necessarily minimal, value of the objective function *ϕ*. This is done in the SM by first investigating the bounded perturbation resilience of an algorithm designed to solve (4.2) and then proactively using such permitted perturbations in order to steer the iterates of such an algorithm toward lower values of the *ϕ* objective function while not loosing the overall convergence to a point in Ψ.

In this paper, we do not investigate superiorization of the extragradient method. We prepare for such an application by proving the bounded perturbation resilience that is needed in order to do superiorization.

### Algorithm 4.1

The basic algorithm:

*Initialization*: $x^{0} \in\Theta$ is arbitrary;

*Iterative step*: Given the current iterate vector $x^{k}$, calculate the next iterate $x^{k+1}$ via

4.5 $$ x^{k+1}=\mathbf{A}_{\Psi}\bigl(x^{k} \bigr). $$

The bounded perturbation resilience (henceforth abbreviated to BPR) of such a basic algorithm is defined next.

### Definition 4.2

An algorithmic operator $\mathbf{A}_{\Psi}: H\rightarrow \Theta$ is said to be bounded perturbations resilient if the following is true. If Algorithm 4.5 generates sequences $\{x^{k}\}_{k=0}^{\infty}$ with $x^{0}\in\Theta$ that converge to points in Ψ, then any sequence $\{y^{k}\}_{k=0}^{\infty}$ starting from any $y^{0}\in\Theta$, generated by

4.6 $$ y^{k+1} = \mathbf{A}_{\Psi}\bigl(y^{k} + \lambda_{k}v^{k}\bigr)\quad\hbox{for all } k \geq0, $$

also converges to a point in Ψ, provided that (i) the sequence $\{v^{k}\}_{k=0}^{\infty}$ is bounded, and (ii) the scalars $\{\lambda_{k}\} _{k=0}^{\infty}$ are such that $\lambda_{k}\geq0$ for all $k \geq0$, and $\sum_{k=0}^{\infty}\lambda_{k}<+\infty$, and (iii) $y^{k}+\lambda_{k}v^{k}\in \Theta$ for all $k \geq0$.

Definition 4.2 is nontrivial only if $\Theta\neq\mathcal{H}$, in which condition (iii) is enforced in the superiorized version of the basic algorithm, see step (xiv) in the ‘Superiorized Version of Algorithm P’ in ([26], p.5537) and step (14) in ‘Superiorized Version of the ML-EM Algorithm’ in ([27], Subsection II.B). This will be the case in the present work.

Treating the extragradient method as the Basic Algorithm $\mathbf {A}_{\Psi}$, our strategy is to first prove convergence of the iterative step (1.2) with bounded perturbations. We show next how the convergence of this yields BPR according to Definition 4.2.

A superiorized version of any Basic Algorithm employs the perturbed version of the Basic Algorithm as in (4.6). A certificate to do so in the superiorization method, see [28], is gained by showing that the Basic Algorithm is BPR. Therefore, proving the BPR of an algorithm is the first step toward superiorizing it. This is done for the extragradient method in the next subsection.

### The BPR of the extragradient method

In this subsection, we investigate the bounded perturbation resilience of the extragradient method whose iterative step is given by (1.2).

To this end, we treat the right-hand side of (1.2) as the algorithmic operator $\mathbf{A}_{\Psi}$ of Definition 4.2, namely, we define, for all $k\geq0$,

4.7 $$ \mathbf{A}_{\Psi}\bigl(x^{k} \bigr)=P_{C}\bigl(x^{k}-\gamma_{k}F \bigl(P_{C}\bigl(x^{k}-\gamma_{k} F \bigl(x^{k}\bigr)\bigr)\bigr)\bigr) $$

and identify the solution set Ψ with the solution set of the variational inequality (1.1) and identify the additional set Θ with *C*.

According to Definition 4.2, we need to show the convergence of the sequence $\{x^{k}\}_{k=0}^{\infty}$ that, starting from any $x^{0}\in C$, is generated by

4.8 $$ x^{k+1}=P_{C}\bigl(\bigl(x^{k}+ \lambda_{k}v^{k}\bigr)-\gamma _{k}F \bigl(P_{C}\bigl(\bigl(x^{k}+\lambda _{k}v^{k} \bigr)-\gamma_{k}F\bigl(x^{k}+\lambda_{k}v^{k} \bigr)\bigr)\bigr)\bigr), $$

which can be rewritten as

4.9 $$ \left \{ \textstyle\begin{array}{l} y^{k}=P_{C}((x^{k}+\lambda_{k}v^{k})-\gamma_{k}F(x^{k}+\lambda _{k}v^{k})), \\ x^{k+1}=P_{C}((x^{k}+\lambda_{k}v^{k})-\gamma_{k}F(y^{k})),\end{array}\displaystyle \right . $$

where $\gamma_{k}=\sigma\rho^{m_{k}}$, $\sigma>0$, $\rho\in(0,1)$ and $m_{k}$ is the smallest nonnegative integer such that

4.10 $$ \gamma_{k}\big\Vert F\bigl(x^{k}+\lambda_{k}v^{k} \bigr)-F\bigl(y^{k}\bigr)\big\Vert \leq\mu \bigl(\big\Vert x^{k}-y^{k} \big\Vert +\lambda_{k}\big\Vert v^{k}\big\Vert \bigr),\quad\mu\in(0,1). $$

The sequences $\{v^{k}\}_{k=0}^{\infty}$ and $\{\lambda_{k}\} _{k=0}^{\infty}$ obey conditions (i) and (ii) in Definition 4.2, respectively, and also (iii) in Definition 4.2 is satisfied.

The next theorem establishes the bounded perturbation resilience of the extragradient method. The proof idea is to build a relationship between BPR and the convergence of the iterative step (1.2).

#### Theorem 4.3

*Assume that Conditions* 3.1-3.3 *hold*. *Assume the sequence* $\{v^{k}\}_{k=0}^{\infty}$ *is bounded*, *and the scalars* $\{\lambda_{k}\}_{k=0}^{\infty}$ *are such that* $\lambda_{k}\geq0$ *for all* $k \geq 0 $, *and* $\sum_{k=0}^{\infty}\lambda_{k}<+\infty$. *Then the sequence* $\{ x^{k}\}_{k=0}^{\infty}$ *generated by* (4.9) *and* (4.10) *converges weakly to a solution of the variational inequality* (1.1).

#### Proof

Take $x^{*}\in \mathit{SOL}(C,F)$. From $\sum_{k=0}^{\infty}\lambda_{k}<+\infty$ and that $\{v^{k}\}_{k=0}^{\infty}$ is bounded, we have

4.11 $$ \sum_{k=0}^{\infty}\lambda_{k}\big\| v^{k}\big\| < +\infty, $$

which means

4.12 $$ \lim_{k\rightarrow\infty}\lambda_{k} \big\| v^{k}\big\| =0. $$

So, we assume $\lambda_{k}\|v^{k}\|\in[0,(1-\mu-\nu)/2)$, where $\nu\in [0,1-\mu)$. Identifying $e_{2}^{k}$ with $\lambda_{k}v^{k}$ in (3.7) and (3.8) and using (3.10), we get

4.13 $$ \begin{aligned} [b]\big\| x^{k+1}-x^{*} \big\| ^{2}={}&\big\| x^{k}-x^{*}\big\| ^{2}+\lambda_{k} \big\| v^{k}\big\| +\lambda_{k}\big\| v^{k}\big\| \big\| x^{k+1}-x^{*}\big\| ^{2}-\big\| x^{k}-y^{k} \big\| ^{2} \\ &-\big\| y^{k}-x^{k+1}\big\| ^{2}+2\bigl\langle x^{k}-\gamma_{k} F\bigl(y^{k}\bigr)-y^{k},x^{k+1}-y^{k} \bigr\rangle . \end{aligned} $$

From $x^{k+1}\in C$, the definition of $y^{k}$ and Lemma 2.2, we have

$$ \begin{aligned} \bigl\langle y^{k}-x^{k}- \lambda_{k}v^{k}+\gamma _{k}F \bigl(x^{k}+\lambda _{k}v^{k} \bigr),x^{k+1}-y^{k}\bigr\rangle \geq0. \end{aligned} $$

So, we obtain

4.14 $$ \begin{aligned}[b] &2\bigl\langle x^{k}-\gamma_{k}F \bigl(y^{k}\bigr)-y^{k},x^{k+1}-y^{k} \bigr\rangle \\ &\quad\leq2\gamma_{k}\bigl\langle F\bigl(x^{k}+\lambda _{k}v^{k}\bigr)-F\bigl(y^{k} \bigr),x^{k+1}-y^{k}\bigr\rangle -2\lambda_{k}\bigl\langle v^{k},x^{k+1}-y^{k}\bigr\rangle . \end{aligned} $$

We have

4.15 $$ \begin{aligned}[b] &2\gamma_{k}\bigl\langle F \bigl(x^{k}+\lambda _{k}v^{k}\bigr)-F \bigl(y^{k}\bigr),x^{k+1}-y^{k}\bigr\rangle \\ &\quad\leq2\gamma_{k}\big\Vert F\bigl(x^{k}+\lambda_{k}v^{k} \bigr)-F\bigl(y^{k}\bigr)\big\Vert \big\Vert x^{k+1}-y^{k} \big\Vert \\ &\quad\leq2\mu\big\Vert x^{k}+\lambda_{k}v^{k}-y^{k} \big\Vert \big\Vert x^{k+1}-y^{k}\big\Vert \\ &\quad\leq2\mu \bigl( \big\Vert x^{k}-y^{k}\big\Vert + \lambda_{k}\big\Vert v^{k}\big\Vert \bigr) \big\Vert x^{k+1}-y^{k}\big\Vert \\ &\quad\leq2\mu\big\Vert x^{k}-y^{k}\big\Vert \big\Vert x^{k+1}-y^{k} \big\Vert +2\mu\lambda _{k}\big\Vert v^{k}\big\Vert \big\Vert x^{k+1}-y^{k}\big\Vert \\ &\quad\leq\mu\big\Vert x^{k}-y^{k}\big\Vert ^{2}+\bigl(\mu+ \lambda_{k}\big\Vert v^{k}\big\Vert \bigr)\big\Vert x^{k+1}-y^{k} \big\Vert ^{2}+\mu^{2}\lambda_{k}\big\Vert v^{k}\big\Vert . \end{aligned} $$

Similar to (3.8), we can show

4.16 $$ \begin{aligned}-2\lambda_{k}\bigl\langle v^{k},x^{k+1}-y^{k}\bigr\rangle \leq \lambda _{k}\big\Vert v^{k}\big\Vert +\lambda_{k}\big\Vert v^{k}\big\Vert \big\Vert x^{k+1}-y^{k}\big\Vert ^{2}. \end{aligned} $$

Combining (4.14)-(4.16), we get

4.17 $$ \begin{aligned}[b] &2\bigl\langle x^{k}-\gamma_{k}F \bigl(y^{k}\bigr)-y^{k},x^{k+1}-y^{k} \bigr\rangle \\ &\quad\leq\mu\big\Vert x^{k}-y^{k}\big\Vert ^{2}+\bigl(\mu+2 \lambda_{k}\big\Vert v^{k}\big\Vert \bigr)\big\Vert x^{k+1}-y^{k} \big\Vert ^{2}+\bigl(1+\mu^{2}\bigr)\lambda_{k}\big\Vert v^{k}\big\Vert \\ &\quad\leq\mu\big\Vert x^{k}-y^{k}\big\Vert ^{2}+(1-\nu)\big\Vert x^{k+1}-y^{k}\big\Vert ^{2}+2\lambda_{k} \big\Vert v^{k}\big\Vert , \end{aligned} $$

where the last inequality comes from $\lambda_{k}\Vert v^{k}\Vert <(1-\mu )/2$ and $\mu<1$. Substituting (4.17) into (4.13), we get

4.18 $$ \begin{aligned}[b] \big\Vert x^{k+1}-x^{\ast} \big\Vert ^{2}\leq{}&\big\Vert x^{k}-x^{\ast}\big\Vert ^{2}-(1-\mu)\big\Vert x^{k}-y^{k} \big\Vert ^{2}-\nu\big\Vert x^{k+1}-y^{k}\big\Vert ^{2}+3\lambda_{k}\big\Vert v^{k}\big\Vert \\ &+\big\Vert x^{k+1}-x^{\ast}\big\Vert ^{2}. \end{aligned} $$

Following the proof line of Theorem 3.7, we get $\{x^{k}\} _{k=0}^{\infty}$ weakly converges to a solution of the variational equality (1.1). □

By using Theorems 3.8 and 4.3, we obtain the convergence rate of the extragradient method with BP.

#### Theorem 4.4

*Assume that Conditions* 3.1-3.3 *hold*. *Assume the sequence* $\{v^{k}\}_{k=0}^{\infty}$ *is bounded*, *and the scalars* $\{\lambda_{k}\}_{k=0}^{\infty}$ *are such that* $\lambda_{k}\geq0$ *for all* $k \geq 0 $, *and* $\sum_{k=0}^{\infty}\lambda_{k}<+\infty$. *Let the sequences* $\{ x^{k}\}_{k=0}^{\infty}$ *and* $\{y^{k}\}_{k=0}^{\infty}$ *be generated by* (4.9) *and* (4.10). *For any integer* $t > 0$, *we have* $y_{t}\in C$ *which satisfies*

4.19 $$ \begin{aligned} \bigl\langle F(x),y_{t}-x \bigr\rangle \leq\frac{1}{2\Upsilon_{t}}\bigl(\big\| x-x^{0} \big\| ^{2}+M(x)\bigr),\quad\forall x\in C, \end{aligned} $$

*where*

4.20 $$ y_{t}=\frac{1}{\Upsilon_{t}}\sum _{k=0}^{t}\gamma_{k}y^{k},\quad \Upsilon_{t}=\sum_{k=0}^{t} \gamma_{k}, $$

*and*

4.21 $$ M(x)=\sup_{k}\bigl\{ \max\bigl\{ \big\| x^{k+1}-y^{k}\big\| ,3\big\| x^{k+1}-x\big\| ^{2}\bigr\} \bigr\} \sum_{k=0}^{\infty}\lambda_{k} \big\| v^{k}\big\| . $$

### Construction of the inertial extragradient methods by BPR

In this subsection, we construct two classes of inertial extragradient methods by using BPR, *i.e*., identifying $e_{i}^{k}$, $k=1,2$, and $\lambda_{k}$, $v^{k}$ with special values.

Polyak [29, 30] first introduced the inertial-type algorithms by using the heavy ball method of the second-order dynamical systems in time. Since the inertial-type algorithms speed up the original algorithms without the inertial effects, recently there has been increasing interest in studying inertial-type algorithms (see, *e.g*., [31–34]). The authors [35] introduced an inertial extragradient method as follows:

4.22 $$ \left\{ \begin{aligned} &w^{k}=x^{k}+ \alpha_{k}\bigl(x^{k}-x^{k-1}\bigr), \\ &y^{k}=P_{C}\bigl(w^{k}-\gamma F \bigl(w^{k}\bigr)\bigr), \\ &x^{k+1}=(1-\lambda_{k})w^{k}+ \lambda_{k}P_{C}\bigl(w^{k}-\gamma F \bigl(y^{k}\bigr)\bigr) \end{aligned} \right . $$

for each $k\geq1$, where $\gamma\in(0,1/L)$, $\{\alpha_{k}\}$ is nondecreasing with $\alpha_{1}=0$ and $0\leq\alpha_{k}\leq\alpha <1$ for each $k\geq1$ and $\lambda,\sigma,\delta>0$ are such that

4.23 $$ \delta>\frac{\alpha{}[(1+\gamma L)^{2}\alpha(1+\alpha )+(1-\gamma ^{2}L^{2})\alpha\sigma+\sigma(1+\gamma L)^{2}]}{1-\gamma^{2}L^{2}} $$

and

$$ 0< \lambda\leq\lambda_{k}\leq\frac{\delta(1-\gamma^{2}L^{2})-\alpha {}[(1+\gamma L)^{2}\alpha(1+\alpha)+(1-\gamma^{2}L^{2})\alpha \sigma +\sigma(1+\gamma L)^{2}]}{\delta{}[(1+\gamma L)^{2}\alpha (1+\alpha )+(1-\gamma^{2}L^{2})\alpha\sigma+\sigma(1+\gamma L)^{2}]}, $$

where *L* is the Lipschitz constant of *F*.

Based on the iterative step (1.2), we construct the following inertial extragradient method:

4.24 $$ \left \{ \textstyle\begin{array}{l} y^{k}=P_{C}(x^{k}-\gamma_{k}F(x^{k})+\alpha _{k}^{(1)}(x^{k}-x^{k-1})), \\ x^{k+1}=P_{C}(x^{k}-\gamma_{k}F(y^{k})+\alpha _{k}^{(2)}(x^{k}-x^{k-1})),\end{array}\displaystyle \right . $$

where

4.25 $$ \alpha_{k}^{(i)}=\left \{ \textstyle\begin{array}{l@{\quad}l} \frac{\beta_{k}^{(i)}}{ \Vert x^{k}-x^{k-1} \Vert }, & \text{if } \Vert x^{k}-x^{k-1} \Vert >1,i=1,2, \\ \beta_{k}^{(i)}, & \text{if } \Vert x^{k}-x^{k-1} \Vert \leq 1.\end{array}\displaystyle \right . $$

#### Theorem 4.5

*Assume that Conditions* 3.1-3.3 *hold*. *Assume that the sequences* $\{\beta_{k}^{(i)}\}_{k=0}^{\infty}$, $i=1,2$, *satisfy* $\sum_{k=1}^{\infty}\beta_{k}^{(i)}<\infty$, $i=1,2$. *Then the sequence* $\{x^{k}\}_{k=0}^{\infty}$ *generated by the inertial extragradient method* (4.24) *converges weakly to a solution of the variational inequality *(1.1).

#### Proof

Let $e_{i}^{k}=\beta_{k}^{(i)}v^{k}$, $i=1,2$, where

4.26 $$ v^{k}=\left \{ \textstyle\begin{array}{l@{\quad}l} \frac{x^{k}-x^{k-1}}{ \Vert x^{k}-x^{k-1} \Vert }, & \text{if } \Vert x^{k}-x^{k-1} \Vert >1,i=1,2, \\ x^{k}-x^{k-1}, & \text{if } \Vert x^{k}-x^{k-1} \Vert \leq1.\end{array}\displaystyle \right . $$

It is obvious that $\Vert v^{k}\Vert\leq1$. So, it follows that $\{e_{i}^{k}\}$, $i=1,2$, satisfy (3.3) from the condition on $\{\beta_{k}^{(i)}\}$. Using Theorem 3.7, we complete the proof. □

#### Remark 4.6

From (3.24), we have $\|x^{k}- x^{k-1}\|\leq1$ for big enough *k*, that is, $\alpha_{k}^{(i)}=\beta_{k}^{(i)}$.

Using the extragradient method with bounded perturbations (4.9), we construct the following inertial extragradient method:

4.27 $$ \left \{ \textstyle\begin{array}{l} y^{k}=P_{C}(x^{k}+\alpha_{k}(x^{k}-x^{k-1})-\gamma_{k}F(x^{k}+\alpha _{k}(x^{k}-x^{k-1}))), \\ x^{k+1}=P_{C}(x^{k}+\alpha_{k}(x^{k}-x^{k-1})-\gamma_{k}F(y^{k})),\end{array}\displaystyle \right . $$

where

4.28 $$ \alpha_{k}=\left \{ \textstyle\begin{array}{l@{\quad}l} \frac{\beta_{k}}{ \Vert x^{k}-x^{k-1} \Vert }, & \text{if } \Vert x^{k}-x^{k-1} \Vert >1,i=1,2, \\ \beta_{k}, & \text{if } \Vert x^{k}-x^{k-1} \Vert \leq1.\end{array}\displaystyle \right . $$

We extend Theorem 4.3 to the convergence of the inertial extragradient method (4.27).

#### Theorem 4.7

*Assume that Conditions* 3.1-3.3 *hold*. *Assume that the sequence* $\{\beta_{k}\}_{k=0}^{\infty}$ *satisfies* $\sum_{k=1}^{\infty }\beta_{k}<\infty$. *Then the sequence* $\{x^{k}\}_{k=0}^{\infty}$ *generated by the inertial extragradient method* (4.27) *converges weakly to a solution of the variational inequality* (1.1).

#### Remark 4.8

The inertial parameter $\alpha_{k}$ in the inertial extragradient method (4.24) is bigger than that of the inertial extragradient method (4.27). The inertial extragradient method (4.24) becomes the inertial extragradient method (4.27) when $\lambda_{k}=1$.

## The extension to the subgradient extragradient method

In this section, we generalize the results of extragradient method proposed in the previous sections to the subgradient extragradient method.

Censor *et al*. [9] presented the subgradient extragradient method (1.4). In their method the step size is fixed $\gamma\in(0,1/L)$, where *L* is a Lipschitz constant of *F*. So, in order to determine the stepsize $\gamma_{k}$, one needs first calculate (or estimate) *L*, which might be difficult or even impossible in general. So, in order to overcome this, the Armijo-like search rule can be used:

5.1 $$ \gamma_{k}\big\Vert F\bigl(x^{k}\bigr)-F\bigl(y^{k} \bigr)\big\Vert \leq\mu\big\Vert x^{k}-y^{k}\big\Vert ,\quad\mu\in(0,1). $$

To discuss the convergence of the subgradient extragradient method, we make the following assumptions.

### Condition 5.1

The mapping *F* is monotone on $\mathcal{H}$, *i.e*.,

5.2 $$ \bigl\langle f(x)-f(y),x-y\bigr\rangle \geq0,\quad\forall x,y\in\mathcal{H}. $$

### Condition 5.2

The mapping *F* is Lipschitz continuous on $\mathcal{H}$ with the Lipschitz constant $L>0$, *i.e*.,

5.3 $$ \big\Vert F(x)-F(y)\big\Vert \leq L\Vert x-y\Vert,\quad\forall x,y\in\mathcal{H}. $$

As before, Censor *et al*.’s subgradient extragradient method [10, Theorem 3.1] can be easily generalized by using some adaptive step rule, for example, (5.1). This result is captured in the next theorem.

### Theorem 5.3

*Assume that Conditions* 3.1, 5.1 *and* 5.2 *hold*. *Then the sequence* $\{x^{k}\}_{k=0}^{\infty}$ *generated by the subgradient extragradient methods* (1.4) *and* (5.1) *weakly converges to a solution of the variational inequality* (1.1).

### The subgradient extragradient method with outer perturbations

In this subsection, we present the subgradient extragradient method with outer perturbations.

#### Algorithm 5.4

The subgradient extragradient method with outer perturbations:

*Step 0*: Select a starting point $x^{0}\in\mathcal{H}$ and set $k=0$.

*Step 1*: Given the current iterate $x^{k}$, compute

5.4 $$ y^{k}=P_{C}\bigl(x^{k}-\gamma_{k}F \bigl(x^{k}\bigr)+e_{1}\bigl(x^{k}\bigr)\bigr), $$

where $\gamma_{k}=\sigma\rho^{m_{k}}$, $\sigma>0$, $\rho\in(0,1)$ and $m_{k}$ is the smallest nonnegative integer such that (see [16])

5.5 $$ \gamma_{k}\big\Vert F\bigl(x^{k}\bigr)-F\bigl(y^{k} \bigr)\big\Vert \leq\mu\big\Vert x^{k}-y^{k}\big\Vert ,\quad\mu\in(0,1). $$

Construct the set

5.6 $$ T_{k}:=\bigl\{ w\in\mathcal{H}\mid\bigl\langle \bigl(x^{k}- \gamma _{k}F\bigl(x^{k}\bigr)+e_{1} \bigl(x^{k}\bigr)\bigr)-y^{k},w-y^{k}\bigr\rangle \leq0\bigr\} , $$

and calculate

5.7 $$ x^{k+1}=P_{T_{k}}\bigl(x^{k}-\gamma_{k}F \bigl(y^{k}\bigr)+e_{2}\bigl(x^{k}\bigr)\bigr). $$

*Step 2*: If $x^{k}=y^{k}$, then stop. Otherwise, set $k\leftarrow(k+1)$ and return to *Step 1.*

Denote $e_{i}^{k}:=e_{i}(x^{k})$, $i=1,2$. The sequences of perturbations $\{e_{i}^{k}\}_{k=0}^{\infty}$, $i=1,2$, are assumed to be summable, *i.e*.,

5.8 $$ \sum_{k=0}^{\infty}\big\Vert e_{i}^{k} \big\Vert < +\infty,\quad i=1,2. $$

Following the proof of Theorems 3.7 and 3.8, we get the convergence analysis and convergence rate of Algorithm 5.4.

#### Theorem 5.5

*Assume that Conditions* 3.1, 5.1 *and* 5.2 *hold*. *Then the sequence* $\{x^{k}\}_{k=0}^{\infty}$ *generated by Algorithm* 5.4 *converges weakly to a solution of the variational inequality* (1.1).

#### Theorem 5.6

*Assume that Conditions* 3.1, 5.1 *and* 5.2 *hold*. *Let the sequences* $\{x^{k}\}_{k=0}^{\infty}$ *and* $\{y^{k}\}_{k=0}^{\infty}$ *be generated by Algorithm* 5.4. *For any integer* $t > 0$, *we have* $y_{t}\in C$ *which satisfies*

5.9 $$ \begin{aligned} \bigl\langle F(x),y_{t}-x \bigr\rangle \leq\frac{1}{2\Upsilon_{t}}\bigl(\big\| x-x^{0} \big\| ^{2}+M(x)\bigr),\quad\forall x\in C, \end{aligned} $$

*where*

5.10 $$ y_{t}=\frac{1}{\Upsilon_{t}}\sum _{k=0}^{t}\gamma_{k}y^{k},\quad \Upsilon_{t}=\sum_{k=0}^{t} \gamma_{k}, $$

*and*

5.11 $$ M(x)=\sup_{k}\bigl\{ \max\bigl\{ \big\| x^{k+1}-y^{k} \big\| ,\big\| x^{k+1}-x\big\| \bigr\} \bigr\} \sum_{k=0}^{\infty } \big\Vert e^{k}\big\Vert . $$

### The BPR of the subgradient extragradient method

In this subsection, we investigate the bounded perturbation resilience of the subgradient extragradient method (1.4).

To this end, we treat the right-hand side of (1.4) as the algorithmic operator $\mathbf{A}_{\Psi}$ of Definition 4.2, namely, we define, for all $k\geq0$,

5.12 $$ \mathbf{A}_{\Psi}\bigl(x^{k} \bigr)=P_{T(x^{k})}\bigl(x^{k}-\gamma_{k}F \bigl(P_{C}\bigl(x^{k}-\gamma_{k} F \bigl(x^{k}\bigr)\bigr)\bigr)\bigr), $$

where $\gamma_{k}$ satisfies (5.1) and

5.13 $$ T\bigl(x^{k}\bigr)=\bigl\{ w\in\mathcal{H}\mid\bigl\langle \bigl(x^{k}-\gamma_{k} F\bigl(x^{k}\bigr) \bigr)-y^{k}, w-y^{k}\bigr\rangle \leq0\bigr\} . $$

Identify the solution set Ψ with the solution set of the variational inequality (1.1) and identify the additional set Θ with *C*.

According to Definition 4.2, we need to show the convergence of the sequence $\{x^{k}\}_{k=0}^{\infty}$ that, starting from any $x^{0}\in \mathcal{H}$, is generated by

5.14 $$ x^{k+1}=P_{T(x^{k}+\lambda_{k}v^{k})}\bigl(\bigl(x^{k}+ \lambda_{k}v^{k}\bigr)-\gamma _{k}F \bigl(P_{C}\bigl(\bigl(x^{k}+\lambda_{k}v^{k} \bigr)-\gamma_{k}F\bigl(x^{k}+\lambda _{k}v^{k} \bigr)\bigr)\bigr)\bigr), $$

which can be rewritten as

5.15 $$ \left \{ \textstyle\begin{array}{l} y^{k} =P_{C}((x^{k}+\lambda_{k}v^{k} )-\gamma_{k}F((x^{k}+\lambda _{k}v^{k})) , \\ T(x^{k}+\lambda_{k}v^{k}) =\{w \in\mathcal{H}\mid\langle((x^{k}+\lambda _{k}v^{k})-\gamma_{k}F(x^{k}+ \lambda_{k}v^{k}))-y^{k}, w-y^{k}\rangle\leq 0\} , \\ x^{k+1} =P_{T(x^{k}+\lambda_{k}v^{k})}((x^{k}+\lambda _{k}v^{k})-\gamma _{k}F (y^{k})), \end{array}\displaystyle \right . $$

where $\gamma_{k}=\sigma\rho^{m_{k}}$, $\sigma>0$, $\rho\in(0,1)$ and $m_{k}$ is the smallest nonnegative integer such that

5.16 $$ \gamma_{k}\big\Vert F\bigl(x^{k}+\lambda_{k}v^{k} \bigr)-F\bigl(y^{k}\bigr)\big\Vert \leq\mu \bigl(\big\Vert x^{k}-y^{k} \big\Vert +\lambda_{k}\big\Vert v^{k}\big\Vert \bigr),\quad\mu\in(0,1). $$

The sequences $\{v^{k}\}_{k=0}^{\infty}$ and $\{\lambda_{k}\} _{k=0}^{\infty}$ obey conditions (i) and (ii) in Definition 4.2, respectively, and also (iii) in Definition 4.2 is satisfied.

The next theorem establishes the bounded perturbation resilience of the subgradient extragradient method. Since its proof is similar to that of Theorem 4.3, we omit it.

#### Theorem 5.7

*Assume that Conditions* 3.1, 5.1 *and* 5.2 *hold*. *Assume the sequence* $\{v^{k}\}_{k=0}^{\infty}$ *is bounded*, *and the scalars* $\{\lambda_{k}\}_{k=0}^{\infty}$ *are such that* $\lambda_{k}\geq0$ *for all* $k \geq 0 $, *and* $\sum_{k=0}^{\infty}\lambda_{k}<+\infty$. *Then the sequence* $\{ x^{k}\}_{k=0}^{\infty}$ *generated by* (5.15) *and* (5.16) *converges weakly to a solution of the variational inequality* (1.1).

We also get the convergence rate of the subgradient extragradient methods with BP (5.15) and (5.16).

#### Theorem 5.8

*Assume that Conditions* 3.1, 5.1 *and* 5.2 *hold*. *Assume the sequence* $\{v^{k}\}_{k=0}^{\infty}$ *is bounded*, *and the scalars* $\{\lambda_{k}\}_{k=0}^{\infty}$ *are such that* $\lambda_{k}\geq0$ *for all* $k \geq 0 $, *and* $\sum_{k=0}^{\infty}\lambda_{k}<+\infty$. *Let the sequences* $\{ x^{k}\}_{k=0}^{\infty}$ *and* $\{y^{k}\}_{k=0}^{\infty}$ *be generated by* (5.15) *and* (5.16). *For any integer* $t > 0$, *we have* $y_{t}\in C$ *which satisfies*

5.17 $$ \begin{aligned} \bigl\langle F(x),y_{t}-x \bigr\rangle \leq\frac{1}{2\Upsilon_{t}}\bigl(\big\| x-x^{0} \big\| ^{2}+M(x)\bigr),\quad\forall x\in C, \end{aligned} $$

*where*

5.18 $$ y_{t}=\frac{1}{\Upsilon_{t}}\sum _{k=0}^{t}\gamma_{k}y^{k},\quad \Upsilon_{t}=\sum_{k=0}^{t} \gamma_{k}, $$

*and*

5.19 $$ M(x)=\sup_{k}\bigl\{ \max\bigl\{ \big\| x^{k+1}-y^{k}\big\| ,3\big\| x^{k+1}-x\big\| ^{2}\bigr\} \bigr\} \sum_{k=0}^{\infty}\lambda_{k} \big\| v^{k}\big\| . $$

### Construction of the inertial subgradient extragradient methods by BPR

In this subsection, we construct two classes of inertial subgradient extragradient methods by using BPR, *i.e*., identifying $e_{i}^{k}$, $k=1,2$, and $\lambda_{k}$, $v^{k}$ with special values.

Based on Algorithm 5.4, we construct the following inertial subgradient extragradient method:

5.20 $$ \left\{ \textstyle\begin{array}{l} y^{k} =P_{C} (x^{k}-\gamma_{k}F(x^{k})+\alpha _{k}^{(1)}(x^{k}-x^{k-1}) )) , \\ T_{k} :=\{w\in\mathcal{H}\mid\langle(x^{k}- \gamma_{k}F(x^{k})+\alpha _{k}^{(1)} (x^{k}-x^{k-1}))-y^{k}, w-y^{k}\rangle\leq0\}, \\ x^{k+1}=P_{T_{k}}(x^{k}-\gamma_{k}F (y^{k})+\alpha _{k}^{(2)} (x^{k}-x^{k-1})), \end{array}\displaystyle \right . $$

where $\gamma_{k}$ satisfies (5.16) and

5.21 $$ \alpha_{k}^{(i)}=\left \{ \textstyle\begin{array}{l@{\quad}l} \frac{\beta_{k}^{(i)}}{ \Vert x^{k}-x^{k-1} \Vert }, & \text{if } \Vert x^{k}-x^{k-1} \Vert >1, i=1,2, \\ \beta_{k}^{(i)}, & \text{if } \Vert x^{k}-x^{k-1} \Vert \leq 1.\end{array}\displaystyle \right . $$

Similar to the proof of Theorem 4.5, we get the convergence of the inertial subgradient extragradient method (5.20).

#### Theorem 5.9

*Assume that Conditions* 3.1, 5.1 *and* 5.2 *hold*. *Assume that the sequences* $\{\beta_{k}^{(i)}\}_{k=0}^{\infty}$, $i=1,2$, *satisfy* $\sum_{k=1}^{\infty}\beta_{k}^{(i)}<\infty$, $i=1,2$. *Then the sequence* $\{x^{k}\}_{k=0}^{\infty}$ *generated by the inertial subgradient extragradient method* (5.20) *converges weakly to a solution of the variational inequality* (1.1).

Using the subgradient extragradient method with bounded perturbations (5.15), we construct the following inertial subgradient extragradient method:

5.22 $$ \left\{ \textstyle\begin{array}{l} w^{k} =x^{k}+\alpha_{k}(x^{k}-x^{k-1} ), \\ y^{k} =P_{C}(w^{k}-\gamma_{k}F (w^{k})), \\ T_{k} :=\{w\in\mathcal{H}\mid\langle(w^{k}- \gamma _{k}F(w^{k}))-y^{k},w-y^{k} \rangle \leq0\}, \\ x^{k+1}=P_{T_{k}}(w^{k}-\gamma_{k}F (y^{k})), \end{array}\displaystyle \right . $$

where $\gamma_{k}=\sigma\rho^{m_{k}}$, $\sigma>0$, $\rho\in(0,1)$ and $m_{k}$ is the smallest nonnegative integer such that

5.23 $$ \gamma_{k}\big\Vert F\bigl(w^{k}\bigr)-F\bigl(y^{k} \bigr)\big\Vert \leq\mu\big\Vert w^{k}-y^{k}\big\Vert ,\quad\mu\in(0,1), $$

and

5.24 $$ \alpha_{k}=\left \{ \textstyle\begin{array}{l@{\quad}l} \frac{\beta_{k}}{ \Vert x^{k}-x^{k-1} \Vert }, & \text{if } \Vert x^{k}-x^{k-1} \Vert >1, i=1,2, \\ \beta_{k}, & \text{if } \Vert x^{k}-x^{k-1} \Vert \leq1.\end{array}\displaystyle \right . $$

We extend Theorem 4.3 to the convergence of the inertial subgradient extragradient method (5.22).

#### Theorem 5.10

*Assume that Conditions* 3.1, 5.1 *and* 5.2 *hold*. *Assume that the sequence* $\{\beta_{k}\}_{k=0}^{\infty}$ *satisfies* $\sum_{k=1}^{\infty }\beta_{k}<\infty$. *Then the sequence* $\{x^{k}\}_{k=0}^{\infty}$ *generated by the inertial subgradient extragradient method* (5.22) *converges weakly to a solution of the variational inequality* (1.1).

## Numerical experiments

In this section, we provide three examples to compare the inertial extragradient method (4.22) (iEG1), the inertial extragradient method (4.24) (iEG2), the inertial extragradient method (4.27) (iEG), the extragradient method (1.2), the inertial subgradient extragradient method (5.20) (iSEG1), the inertial subgradient extragradient method (5.22) (iSEG2) and the subgradient extragradient method (1.4).

In the first example, we consider a typical sparse signal recovery problem. We choose the following set of parameters. Take $\sigma=5$, $\rho=0.9$ and $\mu=0.7$. Set

6.1 $$ \alpha_{k}=\alpha_{k}^{(i)}= \frac{1}{k^{2}}\quad\hbox{if } \big\Vert x^{k}-x^{k-1}\big\Vert \leq1 $$

in the inertial extragradient methods (4.22) and (4.24), and the inertial subgradient extragradient methods (5.20) and (5.22). Choose $\alpha_{k}=0.35$ and $\lambda _{k}=0.8$ in the inertial extragradient method (4.24).

### Example 6.1

Let $x_{0} \in R^{n}$ be a *K*-sparse signal, $K\ll n$. The sampling matrix $A \in R^{m\times n}$ ($m< n$) is stimulated by the standard Gaussian distribution and a vector $b = Ax_{0} + e$, where *e* is additive noise. When $e=0$, it means that there is no noise to the observed data. Our task is to recover the signal $x_{0}$ from the data *b*.

**Figure 1:**
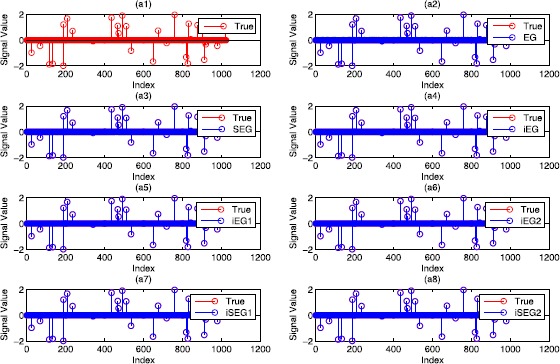
**Comparison of the different methods for sparse signal recovery.** **(a1)** is the true sparse signal, **(a2)**-**(a8)** are the recovered signals vs the true signal by ‘EG’, ‘SEG’, ‘iEG’, ‘iEG1’, ‘iEG2’, ‘iSEG1’ and ‘iSEG2’, respectively.

**Figure 2:**
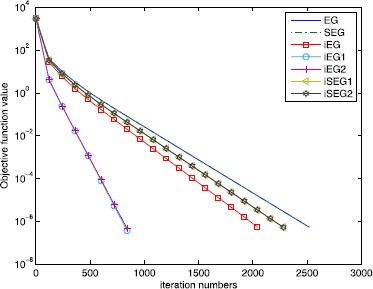
**Comparison of the objective function value versus the iteration numbers of different methods.**

It is well known that the sparse signal $x_{0}$ can be recovered by solving the following LASSO problem [36]:

6.2 $$ \begin{aligned}[b] &\min_{x\in R^{n}} \frac{1}{2}\| Ax - b \|_{2}^{2} \\ &\quad\text{s.t.}\quad \|x\|_{1} \leq t, \end{aligned} $$

where $t >0$. It is easy to see that the optimization problem (6.1) is a special case of the variational inequality problem (1.1), where $F(x) = A^{T}(Ax-b)$ and $C = \{ x \mid \|x\|_{1} \leq t \} $. We can use the proposed iterative algorithms to solve the optimization problem (6.1). Although the orthogonal projection onto the closed convex set *C* does not have a closed-form solution, the projection operator $P_{C}$ can be precisely computed in a polynomial time. We include the details of computing $P_{C}$ in Appendix. We conduct plenty of simulations to compare the performances of the proposed iterative algorithms. The following inequality was defined as the stopping criterion:

$$\big\| x^{k+1} - x^{k} \big\| \leq\epsilon, $$

where $\epsilon>0$ is a given small constant. ‘*Iter*’ denotes the iteration numbers. ‘*Obj*’ represents the objective function value and ‘*Err*’ is the 2-norm error between the recovered signal and the true *K*-sparse signal. We divide the experiments into two parts. One task is to recover the sparse signal $x_{0}$ from noise observation vector *b*, and the other is to recover the sparse signal from noiseless data *b*. For the noiseless case, the obtained numerical results are reported in Table 1. To visually view the results, Figure 1 shows the recovered signal compared with the true signal $x_{0}$ when $K=30$. We can see from Figure 1 that the recovered signal is the same as the true signal. Further, Figure 2 presents the objective function value versus the iteration numbers.

**Table 1:** **Numerical results obtained by the proposed iterative algorithms when** $\pmb{m =240}$ **,** $\pmb{n =1\mbox{,}024}$ **in the noiseless case**

***K*** **-sparse signal**	**Methods**	$\boldsymbol{\epsilon= 10^{-4}}$			$\boldsymbol{\epsilon= 10^{-6}}$		
		***Iter***	***Obj***	***Err***	***Iter***	***Obj***	***Err***
*K* = 20	EG	444	9.7346e-4	0.0080	817	9.6625e-8	7.9856e-5
	SEG	444	9.7272e-4	0.0080	817	9.6555e-8	7.9827e-5
	iEG	374	6.2389e-4	0.0064	675	6.3456e-8	6.4715e-5
	iEG1	159	7.0799e-5	0.0021	263	7.4280e-9	2.2041e-5
	iEG2	158	8.3897e-5	0.0023	273	1.0889e-8	2.6809e-5
	iSEG1	415	8.9563e-4	0.0076	787	2.3571e-7	5.2470e-5
	iSEG2	414	9.2167e-4	0.0077	760	9.1586e-8	7.7275e-5
*K* = 30	EG	1,285	0.0035	0.0281	2,583	3.4535e-7	2.8035e-4
	SEG	1,285	0.0035	0.0281	2,583	3.4534e-7	2.8035e-4
	iEG	1,091	0.0023	0.0227	2,144	2.2732e-7	2.2745e-4
	iEG1	532	3.7493e-4	0.0092	944	3.7522e-8	9.2287e-5
	iEG2	535	3.7961e-4	0.0093	956	4.3181e-8	9.3120e-5
	iSEG1	1,176	0.0031	0.0266	2,351	3.1038e-7	2.6137e-4
	iSEG2	1,176	0.0031	0.0266	2,346	3.1635e-7	2.6784e-4
*K* = 40	EG	1,729	0.0050	0.0405	3,599	5.0237e-7	4.0488e-4
	SEG	1,729	0.0050	0.0405	3,599	5.0228e-7	4.0484e-4
	iEG	1,473	0.0033	0.0328	2,990	3.3182e-7	3.2905e-4
	iEG1	744	5.4838e-4	0.0134	1,361	5.5456e-8	1.3440e-4
	iEG2	745	5.4807e-4	0.0134	1,355	6.4785e-8	1.4191e-4
	iSEG1	1,570	0.0045	0.0384	3,246	4.5079e-7	3.8146e-4
	iSEG2	1,572	0.0045	0.0382	3,244	4.5389e-7	3.8435e-4

**Figure 3:**
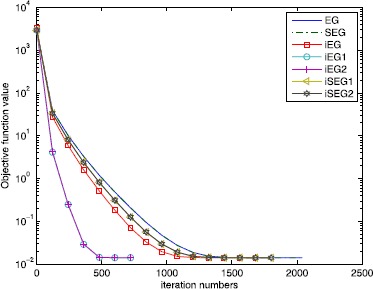
**Comparison of the objective function value versus the iteration numbers of different methods in the noise case of** $\pmb{\beta = 0.02}$ **.**

**Figure 4:**
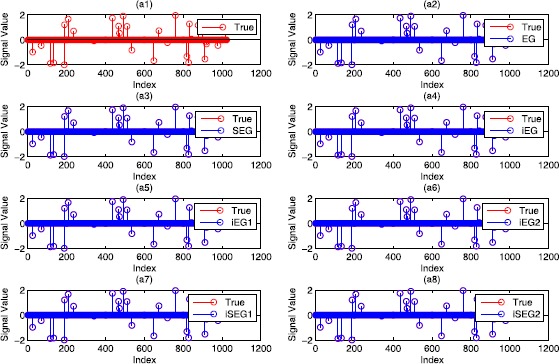
**Comparison of the different methods for sparse signal recovery.** **(a1)** is the true sparse signal, **(a2)**-**(a8)** are the recovered signals vs the true signal by ‘EG’, ‘SEG’, ‘iEG’, ‘iEG1’, ‘iEG2’, ‘iSEG1’ and ‘iSEG2’ in the noise case of $\beta= 0.02$, respectively.

For the noise observation *b*, we assume that the vector *e* is corrupted by Gaussian noise with zero mean and *β* variances. The system matrix *A* is the same as in the noiseless case, and the sparsity level $K=30$. We list the numerical results for different noise level *β* in Table 2. When the noise $\beta= 0.02$, Figure 3 shows the objective function value versus the iteration numbers. Figure 4 shows the recovered signal vs the true signal in the noise case.

**Table 2:** **Numerical results for the proposed iterative algorithms with different noise value** ***β***

**Variances**	**Methods**	$\boldsymbol{\epsilon= 10^{-4}}$			$\boldsymbol{\epsilon= 10^{-6}}$		
		***Iter***	***Obj***	***Err***	***Iter***	***Obj***	***Err***
*β* = 0.01	EG	1,264	0.0092	0.0317	2,192	0.0061	0.0131
	SEG	1,264	0.0092	0.0317	2,192	0.0061	0.0131
	iEG	1,070	0.0081	0.0272	1,812	0.0061	0.0131
	iEG1	519	0.0063	0.0164	788	0.0061	0.0130
	iEG2	516	0.0063	0.0166	786	0.0061	0.0130
	iSEG1	1,156	0.0089	0.0305	1,995	0.0061	0.0131
	iSEG2	1,157	0.0089	0.0304	1,990	0.0061	0.0131
*β* = 0.02	EG	1,274	0.0163	0.0387	2,086	0.0142	0.0272
	SEG	1,274	0.0163	0.0387	2,086	0.0142	0.0272
	iEG	1,070	0.0154	0.0356	1,728	0.0142	0.0272
	iEG1	492	0.0144	0.0300	756	0.0142	0.0272
	iEG2	495	0.0143	0.0300	759	0.0142	0.0272
	iSEG1	1,163	0.0161	0.0378	1,899	0.0142	0.0272
	iSEG2	1,161	0.0161	0.0380	1,895	0.0142	0.0272
*β* = 0.05	EG	1,190	0.1012	0.0749	1,869	0.0991	0.0651
	SEG	1,190	0.1012	0.0749	1,869	0.0991	0.0651
	iEG	996	0.1005	0.0727	1,542	0.0991	0.0650
	iEG1	460	0.0993	0.0677	670	0.0991	0.0650
	iEG2	461	0.0993	0.0675	665	0.0991	0.0650
	iSEG1	1,084	0.1010	0.0742	1,704	0.0991	0.0651
	iSEG2	1,084	0.1010	0.0742	1,704	0.0991	0.0651

### Example 6.2

Let $F:\mathbb{R}^{2} \rightarrow \mathbb{R}^{2}$ be defined by

6.3 $$ F(x,y)=\bigl(2x+2y+\sin(x),-2x+2y+\sin(y)\bigr),\quad\forall x,y \in\mathbb{R}. $$

The authors [37] proved that *F* is Lipschitz continuous with $L=\sqrt{26}$ and 1-strongly monotone. Therefore the variational inequality (1.1) has a unique solution, and $(0,0)$ is its solution.

Let $C=\{x\in\mathbb{R}^{2}\mid e_{1}\leq x\leq e_{2}\}$, where $e_{1}=(-10,-10)$ and $e_{2}=(100,100)$. Take the initial point $x_{0}=(-100,10)\in\mathbb{R}^{2}$. Since $(0,0)$ is the unique solution of the variational inequality (1.1), denote by $D_{k}:=\|x^{k}\|\leq10^{-5}$ the stopping criterion.

### Example 6.3

Let $F: \mathbb{R}^{n} \rightarrow\mathbb{R}^{n}$ defined by $F(x)=Ax+b$, where $A=Z^{T}Z$, $Z=(z_{ij})_{n\times n}$ and $b=(b_{i})\in\mathbb{R}^{n}$, where $z_{ij}\in(0,1)$ and $b_{i}\in(0,1)$ are generated randomly.

It is easy to verify that *F* is *L*-Lipschitz continuous and *η*-strongly monotone with $L=\max(\operatorname{eig}(A))$ and $\eta=\min(\operatorname{eig}(A))$.

Let $C:=\{x\in\mathbb{R}^{n}\mid\Vert x-d\Vert\leq r\}$, where the center

6.4 $$ d\in{}\bigl[(-10,-10,\ldots,-10),(10,10,\ldots,10)\bigr]\subset \mathbb{R}^{n} $$

and radius $r\in(0,10)$ are randomly chosen. Take the initial point $x_{0}=(c_{i})\in\mathbb{R}^{n}$, where $c_{i}\in{}[0,2]$ is generated randomly. Set $n=100$. Take $\rho=0.4$ and other parameters are set the same values as in Example 6.2. Although the variational inequality (1.1) has a unique solution, it is difficult to get the exact solution. So, denote by $D_{k}:=\Vert x^{k+1}-x^{k}\Vert\leq10^{-5}$ the stopping criterion.

**Figure 5:**
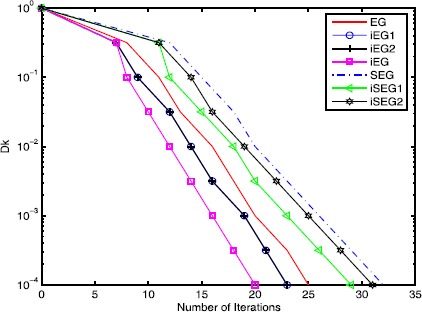
**Comparison of the number of iterations of different methods for Example** **6.2** **.**

**Figure 6:**
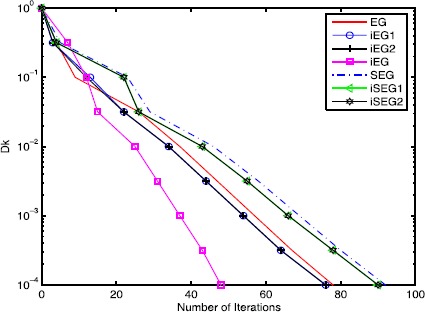
**Comparison of the number of iterations of different methods for Example** **6.3** **.**

From Figures 5 and 6, we conclude: (i) the inertial-type algorithms improve the original algorithms; (ii) the performances of the inertial extragradient methods (4.22) and (4.24) are almost the same; (iii) the inertial subgradient extragradient method (5.20) performs better than the inertial subgradient extragradient method (5.22) for Example 6.1, while they are almost the same for Example 6.2; (iv) the (inertial) extragradient methods behave better than the (inertial) subgradient extragradient methods since the sets *C* in Examples 6.2 and 6.3 are simple, and hence the computation load of the projection onto it is small; (v) the inertial extragradient method (4.22) has an advantage over the inertial extragradient methods (4.22) and (4.24). The reason may be that it takes a bigger inertial parameter $\alpha_{k}$.

## Conclusions

In this research article we study an important property of iterative algorithms for solving variational inequality (VI) problems which is called bounded perturbation resilience. In particular, we focus on extragradient-type methods. This enables us to develop inexact versions of the methods as well as apply the superiorization methodology in order to obtain a ‘superior’ solution to the original problem. In addition, some inertial extragradient methods are also derived. All the presented methods converge at the rate of $O(1/t)$, and three numerical examples illustrate, demonstrate and compare the performances of all the algorithms.

## Appendix

In this part, we present the details of computing a vector $y\in R^{n}$ onto the $\ell_{1}$-norm ball constraint. For convenience, we consider projection onto the unit $\ell_{1}$-norm ball first. Then we extend it to the general $\ell_{1}$-norm ball constraint.

The projection onto the unit $\ell_{1}$-norm ball is to solve the optimization problem

$$ \begin{aligned}[b] &\min_{x\in R^{n}} \frac{1}{2}\| x- y \|_{2}^{2} \\ &\quad\text{s.t.} \quad \|x\|_{1} \leq1. \end{aligned} $$

The above optimization problem is a typical constrained optimization problem, we consider solving it based on the Lagrangian method. Define the Lagrangian function $L(x,\lambda)$ as

$$ L(x,\lambda) = \frac{1}{2}\| x -y \|_{2}^{2} + \lambda\bigl( \|x\|_{1} - 1 \bigr). $$

Let $(x^{*}, \lambda^{*})$ be the optimal primal and dual pair. It satisfies the KKT conditions of

$$ \begin{gathered} 0 \in \bigl(x^{*} - y\bigr) + \lambda^{*} \partial \bigl(\big\| x^{*}\big\| _{1}\bigr), \\ \lambda^{*} \bigl( \big\| x^{*}\big\| _{1} - 1 \bigr) = 0, \\ \lambda^{*} \geq0. \end{gathered} $$

It is easy to check that if $\|y\|_{1} \leq1$, then $x^{*} = y$ and $\lambda^{*} =0$. In the following, we assume $\|y\|_{1} >1$. Based on the KKT conditions, we obtain $\lambda^{*} >0$ and $\|x^{*}\|_{1} = 1$. From the first order optimality, we have $x^{*} = \max\{ |y|-\lambda^{*}, 0 \} \otimes \operatorname{Sign}(y)$, where ⊗ represents element-wise multiplication and $\operatorname{Sign}(\cdot)$ denotes the symbol function, *i.e*., $\operatorname{Sign}(y_{i}) = 1$ if $y_{i} \geq0$; otherwise $\operatorname{Sign}(y_{i}) = -1$.

Define a function $f(\lambda) = \| x(\lambda) \|_{1}$, where $x(\lambda) = S_{\lambda}(y) = \max\{ |y|-\lambda, 0 \}\otimes \operatorname{Sign}(y)$. We prove the following lemma.

### Lemma A.1

*For the function* $f(\lambda)$, *there must exist* $\lambda^{*} >0$ *such that* $f(\lambda^{*}) = 1$.

### Proof

Since $f(0) = \| S_{0}(y) \|_{1} = \|y\|_{1} >1$. Let $\lambda^{+} = \max_{1\leq i \leq n}\{ |y_{i}| \}$, then $f(\lambda^{+}) =0 <1$. Notice that $f(\lambda)$ is decreasing and convex. Therefore, by the intermediate value theorem, there exists $\lambda^{*} >0$ such that $f(\lambda^{*}) = 1$. □

To find $\lambda^{*}$ such that $f(\lambda^{*}) =1$, we follow the following steps.

*Step 1*. Define a vector *y̅* with the same element as $|y|$, which was sorted in descending order. That is, $\overline {y}_{1} \geq\overline{y}_{2} \geq\cdots\geq\overline{y}_{n} \geq0$.

*Step 2*. For every $k= 1,2, \ldots, n$, solve the equation $\sum_{i=1}^{k}\overline{y}_{i} - k \lambda=1$. Stop search until the solution $\lambda^{*}$ belongs to the interval $[\overline{y}_{k+1}, \overline{y}_{k}]$.

In conclusion, the optimal $x^{*}$ can be computed by $x^{*} = \max\{ |y|-\lambda^{*}, 0 \}\otimes \operatorname{Sign}(y)$. The next lemma extends the projection onto the unit $\ell_{1}$-norm ball to the general $\ell _{1}$-norm ball constraint.

### Lemma A.2

*Let* $C_{1} = \{ x \mid \|x\|_{1} \leq1 \}$. *For any* $t>0$, *define a general* $\ell_{1}$-*norm ball constraint set* $C = \{ x \mid \|x \|_{1} \leq t \}$. *Then*, *for any vector* $y\in R^{n}$, *we have*

$$P_{C}(y) = t P_{C_{1}}\biggl(\frac{y}{t}\biggr). $$

### Proof

To compute the projection $P_{C}(y)$ is to solve the optimization problem

$$ \begin{gathered} P_{C}(y) = \arg\min_{x\in R^{n}} \frac{1}{2}\| x-y \|_{2}^{2} \\ \quad\text{s.t.} \quad \| x \|_{1} \leq t. \end{gathered} $$

For any $x\in C$, let $\overline{x} = \frac{x}{t}$, it follows that $\overline{x}\in C_{1}$. The optimal solution $x^{*}$ of the above optimization problem satisfies $x^{*} = P_{C}(y) = t \overline{x}^{*}$, where $\overline{x}^{*}$ is the optimal solution of the optimization problem of

$$ \begin{gathered} \overline{x}^{*} = \arg\min _{\overline{x}\in R^{n}} \frac{1}{2}\bigg\| \overline{x}-\frac{y}{t} \bigg\| _{2}^{2} \\ \quad\text{s.t.} \quad \| \overline{x} \|_{1} \leq1. \end{gathered} $$

It is observed that $\overline{x}^{*}$ is an exact projection onto the closed convex set $C_{1}$. That is, $\overline{x}^{*} = P_{C_{1}}(\frac {y}{t})$. This completes the proof. □
